# Comparison of *Galdieria* growth and photosynthetic activity in different culture systems

**DOI:** 10.1186/s13568-020-01110-7

**Published:** 2020-09-21

**Authors:** Dora Allegra Carbone, Giuseppe Olivieri, Antonino Pollio, Michael Melkonian

**Affiliations:** 1 Laboratory of Biological Oceanography, Stazione Zoologica ‘‘A. Dohrn’’ of Napoli, Villa Comunale, Napoli, I80121 Italy; 2 Bioprocess Engineering, AlgaePARC, Wageningen University and Research, PO Box 16, 6700 AA Wageningen, The Netherlands; 3 Dipartimento di Ingegneria Chimica, dei Materiali e della Produzione Industriale, Università degli Studi di Napoli Federico II, Piazzale Vincenzo Tecchio, 80, 80125 Napoli, Italia; 4 Dipartimento di Biologia, Università degli Studi di Napoli Federico II, Via Cinthia, 26, 80126 Napoli, Italia; 5 Institut für Pflanzenwissenschaften, Universität zu Köln, Zülpicher Str. 47b, 50674 Cologne, Germany; 6 Present Address: Max Planck Institute for Plant Breeding Research, Carl-von-Linne-Weg 10, 50829 Cologne, Germany

**Keywords:** *Galdieria sulphuraria*, photobioreactors, biomass, comparison

## Abstract

In the last years, the acidothermophilic red microalga *Galdieria sulphuraria* has been increasingly studied for industrial applications such as wastewater treatment, recovery of rare earth elements, production of phycobilins. However, even now it is not possible an industrial cultivation of this organism because biotechnological research on *G. sulphuraria* and allied species is relatively recent and fragmented. Having in mind a possible scale-up for commercial applications, we have compared the growth and photosynthetic performance of *G. sulphuraria* in four suspended systems (Inclined bubble column, Decanter Laboratory Flask, Tubular Bioreactor, Ultra-flat plate bioreactor) and one immobilized system (Twin Layer Sytem). The results showed that *G. sulphuraria* had the highest growth, productivity and photosynthetic performance, when grown on the immobilized system, which also offers some economics advantages.

## Keypoints

- Comparison of different microalgal cultivation systems (suspended and immobilized)
- Analysis of growth and photosynthetic performance
- Immobilized cultivation on the Twin layer system showed the best performance with respect to growth and photosynthesis

## Introduction

*Cyanidiophyceae* are a class of red microalgae living in extreme environments (Albertano et al. 2000; Pinto et al. 2003; Yoon et al. 2004). They prevalently thrive in geothermal volcanic areas at temperatures around 40 °C and at high sulfuric acid concentrations, with ambient pH values between 1 and 3 (Albertano et al. 2000; Pinto et al. 2007; Toplin et al.^2008^; Castenholz and Mcdermott 2010; Ciniglia et al. 2014; Ciniglia et al. 2017)

These extreme environmental conditions strongly limit contaminations that are prevalent in open microalgal mass cultivation systems. In consequence, these organisms are of considerable interest for commercial applications (Carfagna et al. 2018; Carbone et al. 2019).

*Cyanidiophyceae* are divided into three genera, *Cyanidium*, *Galdieria*, and *Cyanidioschyzon* (Gross 2000; Heilmann and Gross 2001; Ciniglia et al. 2004; Del Mondo et al.^2019^) but only *Galdieria* is known to grow heterotrophically, also achieving a higher biomass density (Gross et al. 1998; Gross and Schnarrenberger 1995; Graziani et al. 2013, Vítová et al. 2016); therefore it is considered a promising candidate for industrial applications.

Indeed, *Galdieria* has been the subject of different studies in algal biotechnology. It was used for wastewater treatment (Ju et al. 2016; Henkanette-Gedera et al. 2016; da Silva et al. 2016; Carbone et al. 2018; Galasso et al. 2019; Alalwan et al. 2019; Sosa-Hernández et al. 2019) and for recovery of rare earth elements (Minoda et al. 2015). Moreover, this organism produces high levels of phycobiliproteins that are used in diverse medical and cosmetic products (Schmidt et al. 2005; Graverholt and Eriksen 2007; Sørensen et al. 2013; Eriksen 2018) and in different compounds with antioxidant properties (Carfagna et al. 2016).

However, biotechnological research on *Galdiera* is relatively recent. The data around the growth of this microalga are still fragmentary and even now it is not possible an industrial cultivation of this organism.

Therefore, having in mind a possible scale-up and commercial applications of *G. sulphuraria,* in this paper, the growth and the photosynthetic performance of this microalga were systematically compared in five different types of cultivation systems (one immobilized and four suspended) at the same conditions of temperature and irradiance.

## Materials and methods

### Algal strain and stock cultures

*Galdieria sulphuraria* strain 064 from ACUF collection (D’elia et al. 2018http://www.acuf.net) was chosen. The stock culture was cultivated in *Galdieria* medium (Gross and Schnarrenberger 1995) acidified by sulfuric acid at pH 1.5. Stock cultures were grown in 1 l Erlenmeyer flasks and were exposed to an adaptive light intensity of 30 μmol photons m^−2^ s^−1^ with a light/dark cycle of 14/10 h. The temperature was 35 °C.

### Analysis of growth

We consider several parameters to analyse growth. These parameters are depending variables of time, the only independent variable. Some depending variables, denoted with the term “specific”, are normalized by dividing by the initial values, to take the different inocula into account (conversely, the non-normalized depending variables can be obtained multiplying the normalized ones by the initial values). We explicitly observe that normalization is necessary because Twin-Layer S needs inocula concentrations very different from those used for suspended systems.

The considered variables are: coefficient of determination, specific weight increase, specific light yield, growth rate.

### Coefficient of determination

The coefficient of determination (r^2^) is a measure of how close the data are to the regression line. It was used to compare the different bioreactor systems.

### Specific weight increase (SWI)

The specific weight increase (SWI) was used to analyse the trend of growth in the different bioreactors.

This is the formula defining SWI:

$$SWI\left( t \right) = \frac{w\left( t \right) - w\left( 0 \right)}{w\left( 0 \right)}$$

where *w(t)* is the dry weight at day *t* (more exactly*, t* is the number of the day when the sampling is taken and measured) and *w(0)* the dry weight at day *0* (g).

### Specific light yield (SLY)

To consider the light energy necessary for the growth, we used the standard light yield and normalized it. The formula for the specific light yield (SLY) (photons mol^−1^) is the following:

$$SLY\left( t \right) = \frac{SWI\left( t \right)}{A*t*s*pm}$$

where *SWI(x)* is the specific weight increase, *A* the area of surface of the bioreactor exposed to the light (m^2^), *t* is the number of days, s is the number of seconds of illumination per day (s) (in our case, this number, 50,400, is obtained multiplying the number of illuminations hours, 14, by the number of seconds in a hour, 3600), *pm* is the number of the given moles of photosynthetically active photons per second and per square meter (photons mol s^−1^ m^−2^) (in our case, *pm* is the number of the given PAR, 100, multiplied by 10^−6^).

### Growth rate (GR)

The growth rate in the time period is calculated thanks to the growth rate GR (day^−1^) with this formula:

$$GR\left( t \right) = \frac{{Ln\frac{{w\left( {t + h} \right)}}{w\left( t \right)}}}{{\begin{array}{*{20}c} { } \\ h \\ \end{array} }}$$

where Ln is the natural logarithm, *w(t+h)* is the dry weight at day *t+ h, w(*t) is the dry weight at day *t*, *h* is the number of days between two consecutive measures (in our case, *h* is equal to 3).

### Determination of biomass

In liquid cultivation systems, 2 ml of the culture was harvested every three days in triplicate with a sterile syringe for dry mass determinations and then filtered on a polycarbonate disc using a vacuum pump. In Twin Layer System, the polycarbonate discs were taken off from the bioreactor and biomass in the inoculated area was considered, while the rest was scraped off.

All samples were lyophilized in a freeze dryer for two hours and weighed with an analytical balance (Sartorius Bovenden, Germany).

## Analysis of the photosynthetic state of microalgae

Pigment concentration: microalgae were harvested and lyophilised then they were mixed with quartz sand to obtain homogeneous powder.

Photosynthetic pigments were extracted overnight with acetone (Costache et al. 2012). Chlorophyll a and carotenoids were analysed by spectrophotometry (Shimadzu UV-2450) (Tomitani et al. 1999).

### Pigment concentration

Different formulae were considered to compare the photosynthetic state of each culture.

These equations were used:

$$Chl\,a = 11.75*\left( {A662} \right) - 2.350*\left( {A645} \right)$$

$$Carotenoids = \frac{{1000*\left( {A470} \right) - \left( {2.270 \,Chl\,a} \right)}}{227}$$

where *Chl a* is the concentration of chlorophyll a (mg l^−1^), *Carotenoids* is the concentration of total carotenoids (mg l^−1^), and A is the absorbance at different wavelengths (662, 645,470 nm) (Costache et al. 2012).

### Specific pigments increase

The trend of pigment concentration during growth tests was calculated according to the formula:

$$SP\left( t \right) = \frac{p\left( t \right) - p\left( 0 \right)}{p\left( 0 \right)}$$

where *p(t)* is the concentration of the pigment (chlorophyll or carotenoids) at time t (mg ml^−1^ for liquid systems and g m^−2^ for Twin Layer System) and p(0) is the concentration of the pigment at time 0 (mg ml^−1^ for liquid systems and g m^−2^ for Twin Layer System), obtained by the previous formulae.

### Normalized photosynthesis efficiency (NPE)

NPE is the efficiency of solar light energy captured and stored in biomass. Therefore it is used to estimate the productivity. We normalized the standard formula for photosynthetic efficiency (De Vree et al. 2015) by using the dry weight at time 0. The formula for NPE (g^−1^) is the following:

$$NPE\left( t \right) = \frac{{\Delta H_{C}^{0} *\left( { w\left( {t + h} \right) - w\left( t \right)} \right)}}{w\left( 0 \right)*h*A*s*pm*N*e}$$

where $\Delta H_{C}^{0}$ is the standard enthalpy of combustion (22.5 kJ g^−1^), *w(x + h*) the biomass dry weight at day *t + h* (g), *w(x*) the biomass dry weight at day t (g), *w(0)* the biomass dry weight at time 0 (g), *h* the number of days between two consecutive measures (in our case, *h* is equal to 3), *A* the area of surface of the bioreactor exposed to the light (m^2^), *s* is the number of seconds of illumination per day (s) (in our case, this number, 50.400, is obtained multiplying the number of illuminations hours, 14, by the number of seconds in a hour, 3.600), *pm* is the number of the given moles of photosynthetically active photons per second and per square meter (photons mol s^−1^ m^−2^) (in our case, *pm* is the number of the given PAR, 100, multiplied by 10^−6^), *N* is the Avogadro number, *e* is the approximate energy of a photon of 400 nm 173 wave length (kJ) (this value is around 4 * 10^−22^).

In this formula, we normalized by w(0) to highlight the relevant differences between the TL-S system and suspended systems. Moreover, we acknowledge that it can also be significant to normalize by dividing by w(t) be evidence possible differences between consecutive measurements.

This variable is linked to the productivity of bioreactors and represents the efficiency with which solar energy is captured and stored in biomass (De Vree et al. 2015).

### Photobioreactors and bottle design set up

**Fig. 1:**
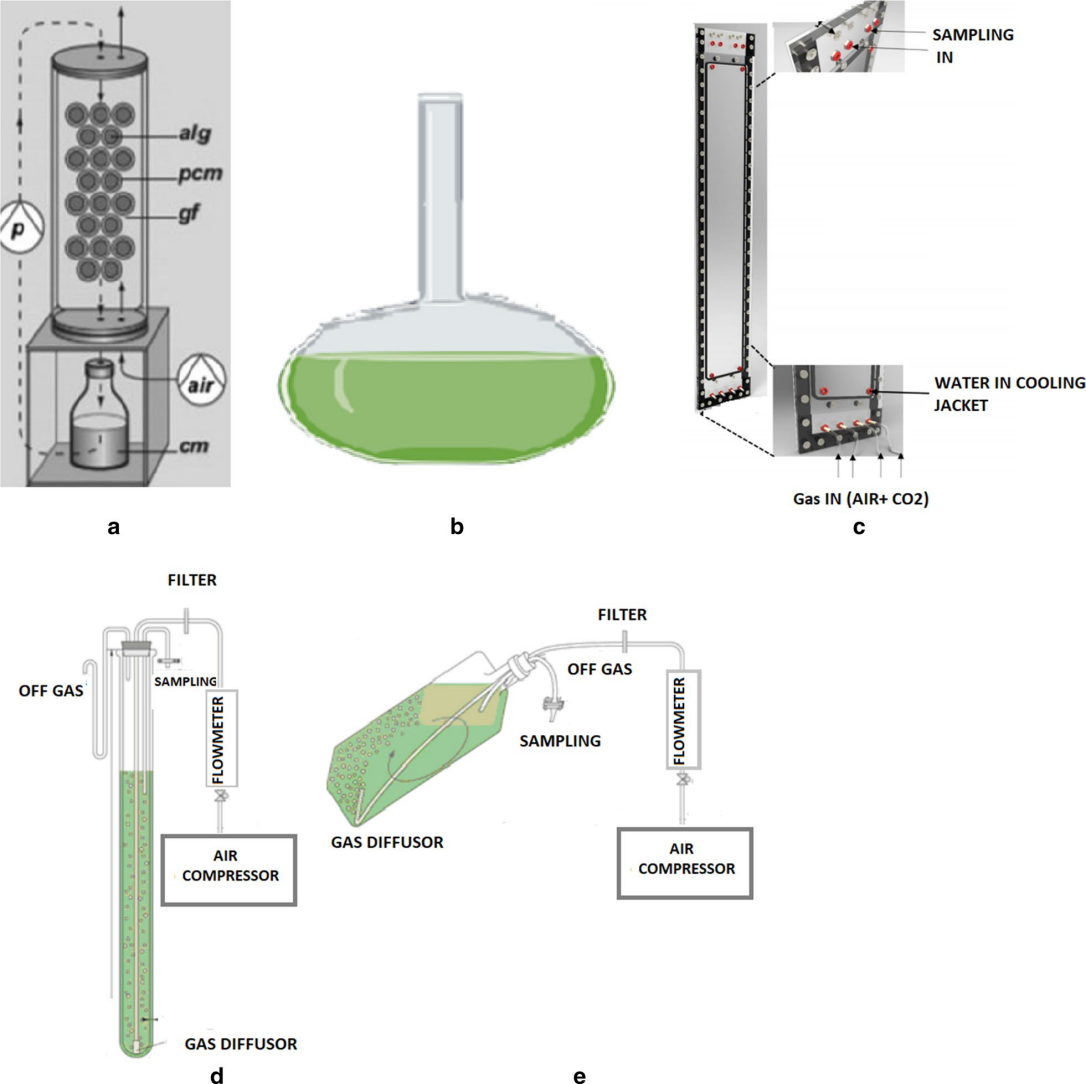
**a** Twin-layer system (Twin Layer-S): *alg* immobilized microalgae, *pcm* polycarbonate membrane as a carrier for microalgae, *gf* glass fiber material, *air* membrane pump for air supply, *cm* culture medium (figure reproduced and modified from Carbone et al. 2017a, b); **b** Decanter (Decanter-LF); **c** A side view of Ultraflate photobioreactor (UP-B) (Gifuni et al. 2018**d** Tubular bioreactor (Tubular-B); **e** Inclined bubble column (Inclined Bubble-C) (Olivieri et al. 2012)

The experiments were set up at the same light intensity of 100 μmol photons m^−2^ s ^−1^ with a light/dark cycle of 14/10 h in presence of atmospheric CO_2_ and at constant temperature of 35 °C. The systems used for the experiments are four suspension systems and one where cells are immobilized on photobioreactor (Fig. 1). In suspension systems the volume of the culture is invariable, because after the sampling of 2 ml water loss was replaced and the growth was influenced only very weakly. At the beginning of the experiment, the culture had an optical density of 0.4 and a dry weight of 0.4 g/l while in the Twin Layer System the culture had a dry weight of 20 g m^−2^.

- The Twin Layer System (Twin Layer-S) consisted of an immobilized photobioreactor where microalgae are inoculated on a polycarbonate disk that is attached on a hydrophilic substrate by self-adhesion, separating the algal biomass from the bulk of the medium (Nowack et al. 2005; Melkonian and Podola 2010; Li et al. 2017). The algae were placed on the Twin Layer-S only when the liquid culture achieved a sufficient density in suspension (optical density around 0.4). Then the algae were harvested by centrifugation for 30 minutes at 2000 rpm (Sorvall, RC5C), filtered onto polycarbonate membranes (PC40, 0.4 μm pore size, 25 mm diameter, Whatman, Dassel, Germany) and subsequently attached to the hydrophilic substrate (Fig. 1a). This system was chosen because it reproduces the natural habitat of this species, generally growing on substrates like soil and rocks (Gross et al. 1998; Ciniglia et al. 2004; Pinto et al. 2007).
- The Decanter laboratory flask (Decanter-LF) had a lighted surface area of 10,201 cm^2^ and was placed on a platform shaker with at a speed of 50 rpm. The total volume of the Decanter-LF was 1000 ml and the working volume was 250 ml (Fig. 1b). The Decanter-LF is not a bioreactor and there isn’t air flux but it was selected because it is the most common system used in *Galdieria* growth test (e.g., Iovinella et al. 2020).
- The Ultra-flat plate bioreactor (Flat-UPB) had a lighted surface area of 715 cm^2^ and was composed of three plexiglass panels spaced by two silicone sheets. Four 1 mm orifices from the bottom of the photobioreactor aerated the system with a gas stream. The total volume was 700 ml and working volume was 400 ml (Fig. 1c). This reactor was chosen because it has a high surface area to volume ratio (Gifuni et al. 2018; Zuccaro et al. 2020).
- The Tubular bioreactor (Tubular-B) was a glass column photobioreactor, with a lighted surface area of 275 cm^2^ and a glass pipe with a membrane pump equipped with a sterile filter at the bottom of the column aerating the system. The total volume was 350 ml, the working volume was 200 ml (Fig. 1d). This type of system was chosen because mixing of the suspension is optimal (Aslanbay Guler et al. 2019; Carbone et al. 2019; Dupré et al. 2020).
- The Inclined bubble column bioreactor (Inclined Bubble-C) was a prism of 2 litres with a rectangular base and a lighted surface area of 300 cm^2^. On the bottom of the bioreactor, the gas stream was sparged by multiple orifices of a Teflon tube. The working volume was 400 ml (Fig 1e). This system was chosen because it has a good ratio between the photic and the dark zone and the microalgae are not exposed to an excess of light or darkness (Olivieri et al. 2013).

## Results

### Algal growth in different cultivation systems

During the experiment, *G. sulphuraria* showed differences in growth in the cultivation systems.

**Fig. 2:**
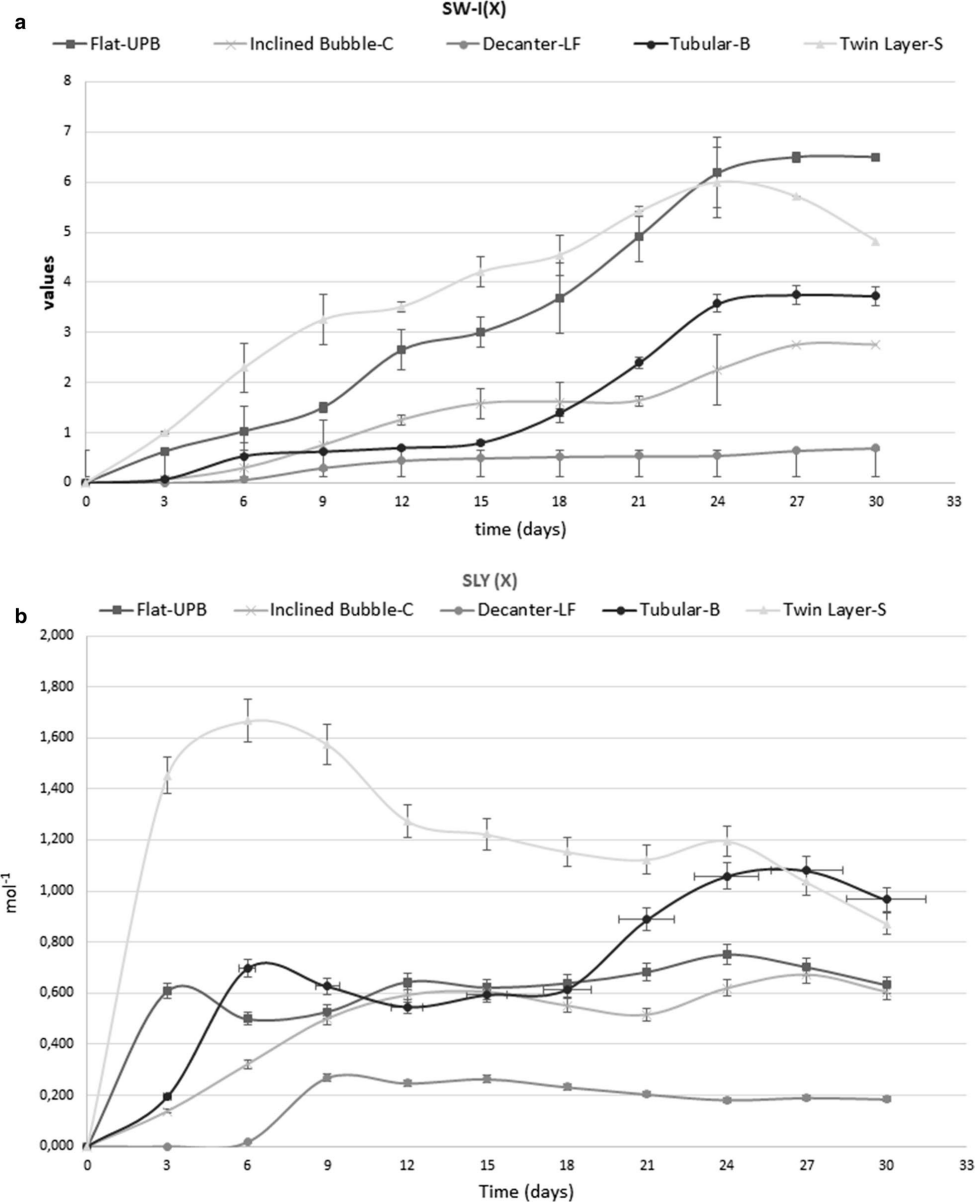
**a** Specific weight increase (SWI) values in the different systems during 30th days. **b** Specific light yield (SLY) values in the different systems during 30th days

The slowest growth was observed in Decanter-LF where, at the end of the experiment, *G. sulphuraria* was only at the beginning of the exponential growth phase (Fig. 2; Table 1).

**Table 1:** Growth parameters of microalgae in different systems

DAYS	Decanter laboratory flask (DLF)	Inclined bubble column (IB-C)	Tubular bioreactor (T-B)	Ultra-flat plate bioreactor (UP-B)	Twin layer system (TW-S)
Specific weight increase (SWI)					
0	0	0	0	0	0
3	0	0.0625	0.075	0.625	1
6	0.0625	0.517	0.5357	1.025	2.29
9	0.305	0.7	0.625	1.5	3.25
12	0.45	0.903	0.7	2.65	3.51
15	0.5	1.033	0.8	3	4.2
18	0.52	1.044	1.4	3.69	4.84
21	0.541	1.0566	2.39	4.9	5.4
24	0.55	1.3	3.57	6.1	6.2
27	0.65	1.5	3.75	6.5	5.7
30	0.7	1.5	3.72	6.5	4.7
Specific light yield (SLY) Mol^−1^					
0	0	0	0	0	0
3	0	0.137	0.194	0.608	1.4
6	0.017	0.321	0.697	0.498	1.66
9	0.269	0.501	0.626	0.527	1.57
12	0.248	0.593	0.546	0.644	1.27
15	0.265	0.606	0.593	0.622	1.22
18	0.233	0.551	0.615	0.639	1.15
21	0.205	0.516	0.887	0.683	1.12
24	0.185	0.620	1.05	0.752	1.19
27	0.191	0.670	1.08	0.702	1.03
30	0.181	0.606	0.966	0.632	0.87
Growth rate (GR) day^−1^					
0	0	0	0	0	0
3	0.020	0.020	0.024	0.140	0.221
6	0.068	0.06	0.119	0.073	0.178
9	0.034	0.101	0.018	0.07	0.100
12	0.011	0.085	0.015	0.126	0.083
15	0.006	0.044	0.019	0.03	0.019
18	0.003	0.0035	0.095	0.053	0.047
21	0.0018	0.0035	0.115	0.077	0.020
24	0.0020	0.0694	0.099	0.06	0.029
27	0.02	0.0477	0.0136	0.014	− 0.014
30	0.09	0	− 0.007	0	− 0.04

In the Inclined Bubble-C, the microalgae achieved the stationary growth phase on day 27 but the growth performance was lower than those observed in the others bioreactors (Fig. 2; Table 1).

Also, the Tubular -B and Flat-UPB achieved the stationary growth phase on the day 27 but showed a different behaviour (Fig. 2; Table 1). Indeed, the flat-UPB showed highest values of SWI compared to the other suspension-based bioreactors (around 6.5), while the SLY values were lower than those in the Tubular-B, in which the maximum value was around 0.752 mol^−1^ on day 24 (Fig. 2; Table 1). The maximum GR value was similar in the two bioreactors (approximately 140 day^−1^; Table 1).

**Fig. 3:**
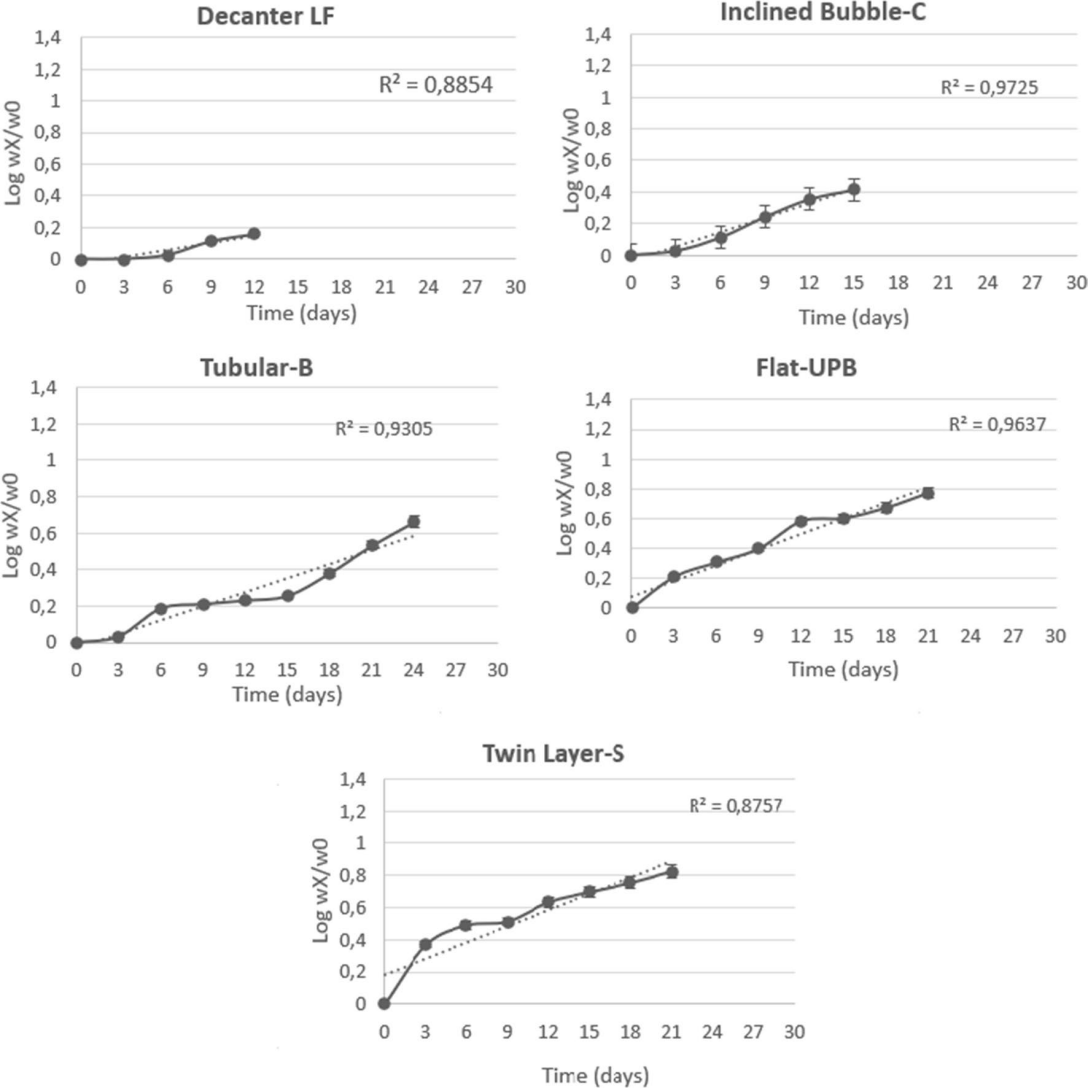
Biomass trends and equation of the line and R^2^ values in the different systems

In the Twin Layer-S, the SWY maximum values were similar to those of the Flat-UPB while SLY were significantly higher during the first 21 days of cultivation than the values obtained in the other bioreactors (maximally 1.7 mol^−2^ on day 6, Fig. 2; Table 1). The values declined only in the last time of the tests when SLY values fell below 1.0 (Fig. 2b). Also, the maximum GR was higher in the Twin Layer-S than in the other bioreactors (0.222 day^−1^). Instead, the r^2^ was the lowest of all photobioreactors (Fig. 3; Table 1).

### Photosynthetic activity

#### Characterization of photosynthetic pigments

The photosynthetic pigments were analysed at the same time as biomass growth. Specifically, chlorophyll a and carotenoids were considered.

**Fig. 4:**
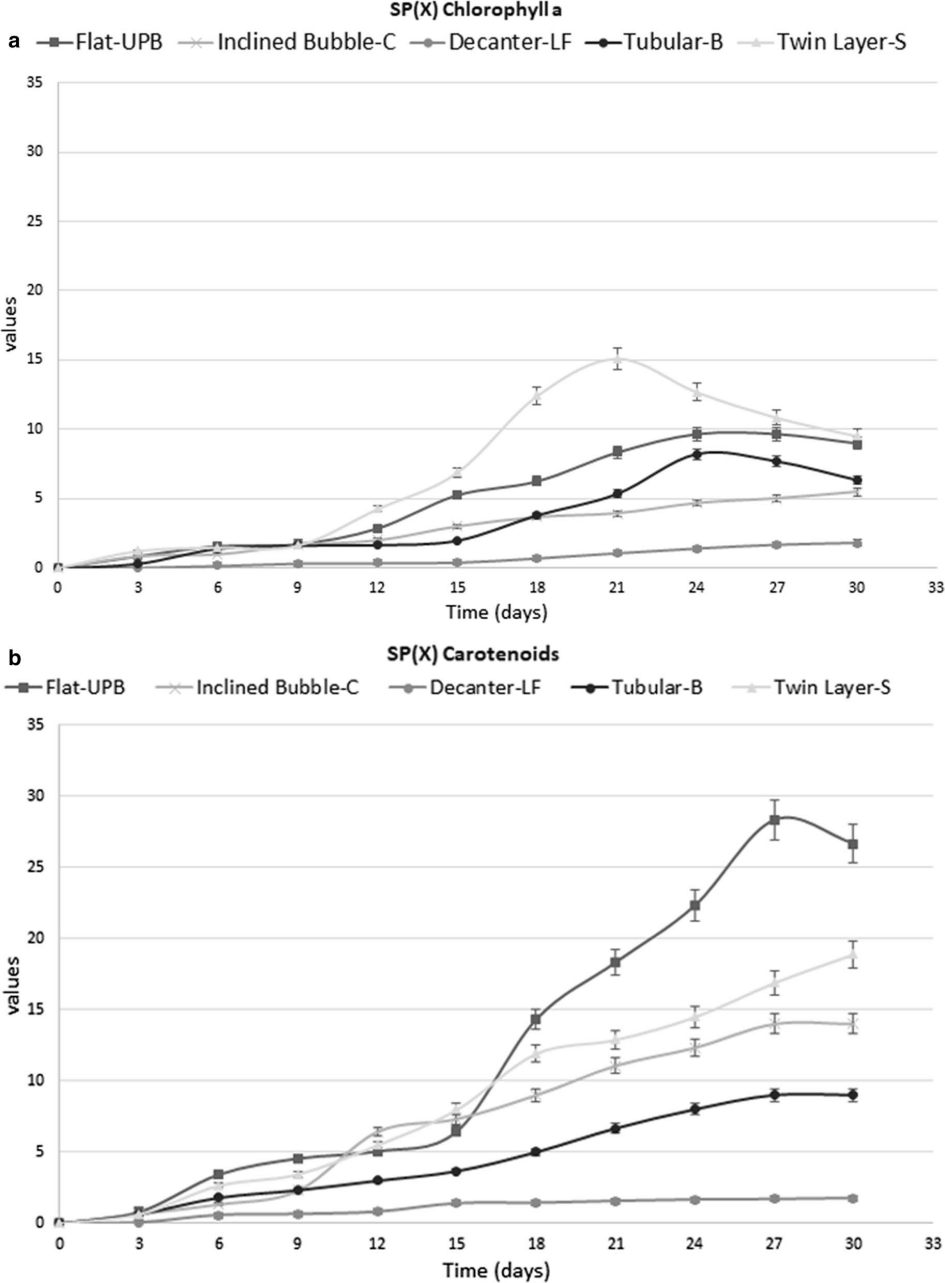
**a** Specific chlorophyll a increase in different systems during the course of experiment. **b** Specific total carotenoids increase in different systems during the course of experiment

As in the case of biomass growth, the Decanter-LF showed the lowest chlorophyll SP levels (Fig. 4a; Table 2). Indeed, the SP(*x*) achieved a maximum value of 2 only on the last day of the experiment.

**Table 2:** Specific increase of pigments in different systems

DAYS	Decanter laboratory flask (DLF)	Inclined bubble column (IB-C)	Tubular bioreactor (T-B)	Ultra-flat plate bioreactor (UP-B)	Twin layer system (TW-S)
Specific pigment increase (SP) chlorophyll a					
0	0	0	0	0	0
3	0.04	0.86	0.32	0.86	1.2
6	0.16	1	1.4	1.6	1.49
9	0.33	1.66	1.62	1.73	1.62
12	0.36	2	1.66	2.86	4.26
15	0.4	3	1.98	5.26	6.89
18	0.7	3.66	3.8	6.26	12.4
21	1.06	3.9	5.3	8.2	15.1
24	1.4	4.66	8.2	9.6	12.68
27	1.66	5	7.6	9.6	10.8
30	1.8	5.4	6.3	9	9.5
Specific pigment increase (SP) carotenoids					
0	0	0	0	0	0.64
3	0.033	0.66	0.66	0.81	2.65
6	0.56	1.3	1.8	3.43	3.45
9	0.63	2.3	2.3	4.5	5.49
12	0.8	6.4	3	5	7.98
15	1.4	7.3	3.6	6.4	11.92
18	1.43	9	5	14.4	12.3
21	1.56	11	6.6	18.3	14.23
24	1.66	12.3	8	22.33	16.2
27	1.7	15	9	28.3	18.9
30	1.76	14	9	26.6	18.8

In the Inclined Bubble-C, chlorophyll a achieved the maximum SP value on day 30 (around 5.4) where in the Tubular B, the maximum SP value was observed on day 27 (around 7.5 Fig. 4a; Table 2).

Compared to the other suspension-based cultivation systems, the flat-UPB showed the highest SP(*x*) level of chlorophyll a (around 10) on day 24, and then decreasing around 9 on day 30 (Fig. 4a; Table 2).

In the Twin Layer-S, the chlorophyll a SP maximum value was on day 21, when it reached a value of 15 (Fig. 4a; Table 2).

**Fig. 5:**
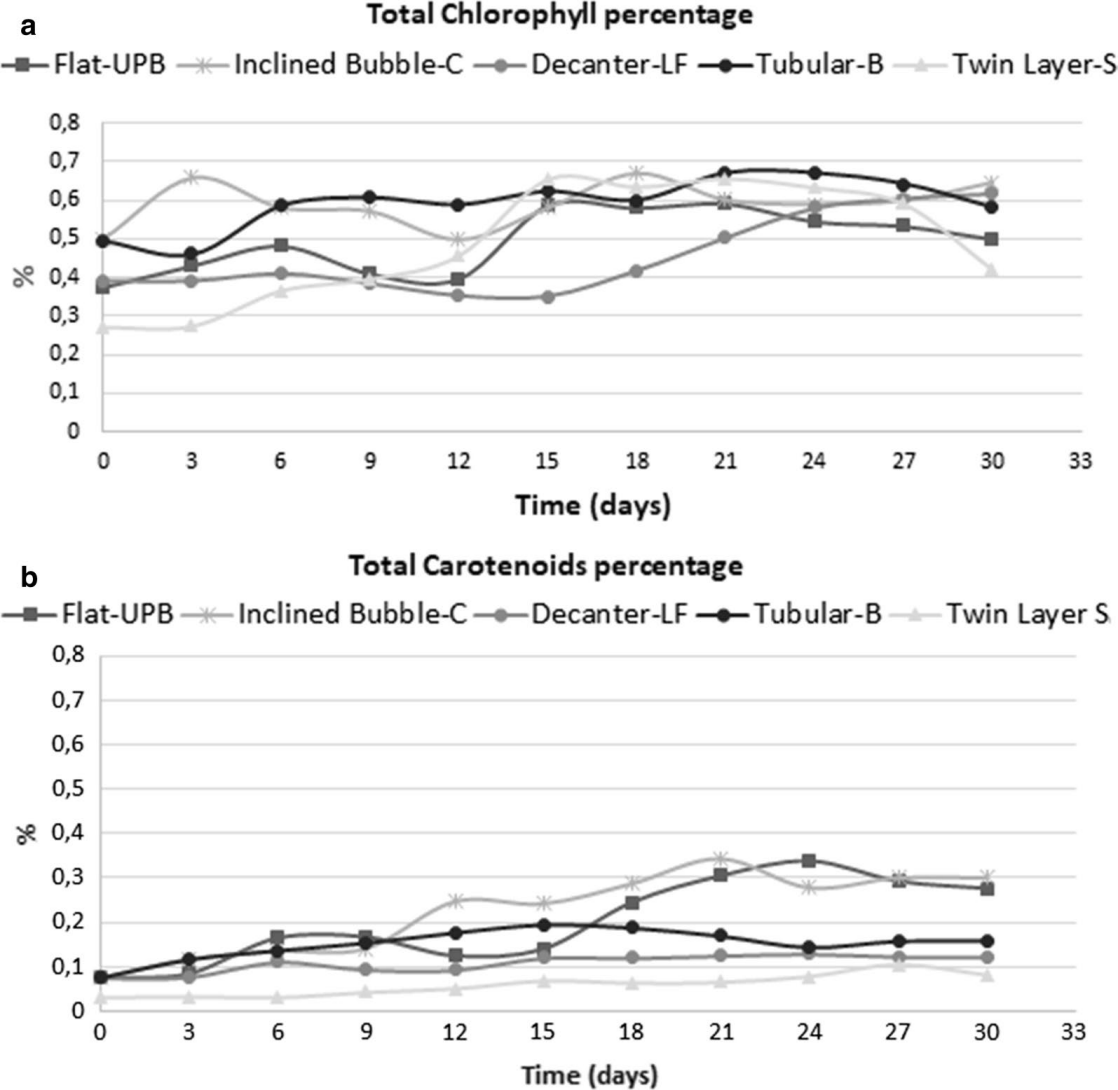
**a** Chlorophyll a percentage during the course of the experiments. **b** Carotenoids percentage during the course of the experiments

In all systems, the chlorophyll a percentage was around 0.6% of the total weight(Fig. 5a).

The carotenoids had a different trend from chlorophyll a except for the Decanter-LF, in which SP values were similar to those of chlorophyll a (around 1) but the maximum percentage value was 0.3% of total weight (Figs. 4b, 5b; Table 2).

The SP values for carotenoids were higher in the Inclined Bubble-C(14) than in the Tubular-B (9) and consequently also the maximum percentage value was higher in the Inclined Bubble-C (0.3% and 0.15% respectively) (Figs. 4b, 5b; Table 2).

In the Flat-UPB, the percentage maximum value was around 0.3% (day 27) and the SP values were higher than in the other suspension-based photobioreactors, achieving a maximum value around 28 on the day 27.

In the Twin Layer-S, SP for carotenoids displayed a lower value than that in the Flat-UPB (around 20 on day 30) and the percentage maximum value was only 0.1% (Figs. 4b, 5b; Table 2).

#### Normalized photosynthesis efficiency (NPE)

**Fig. 6:**
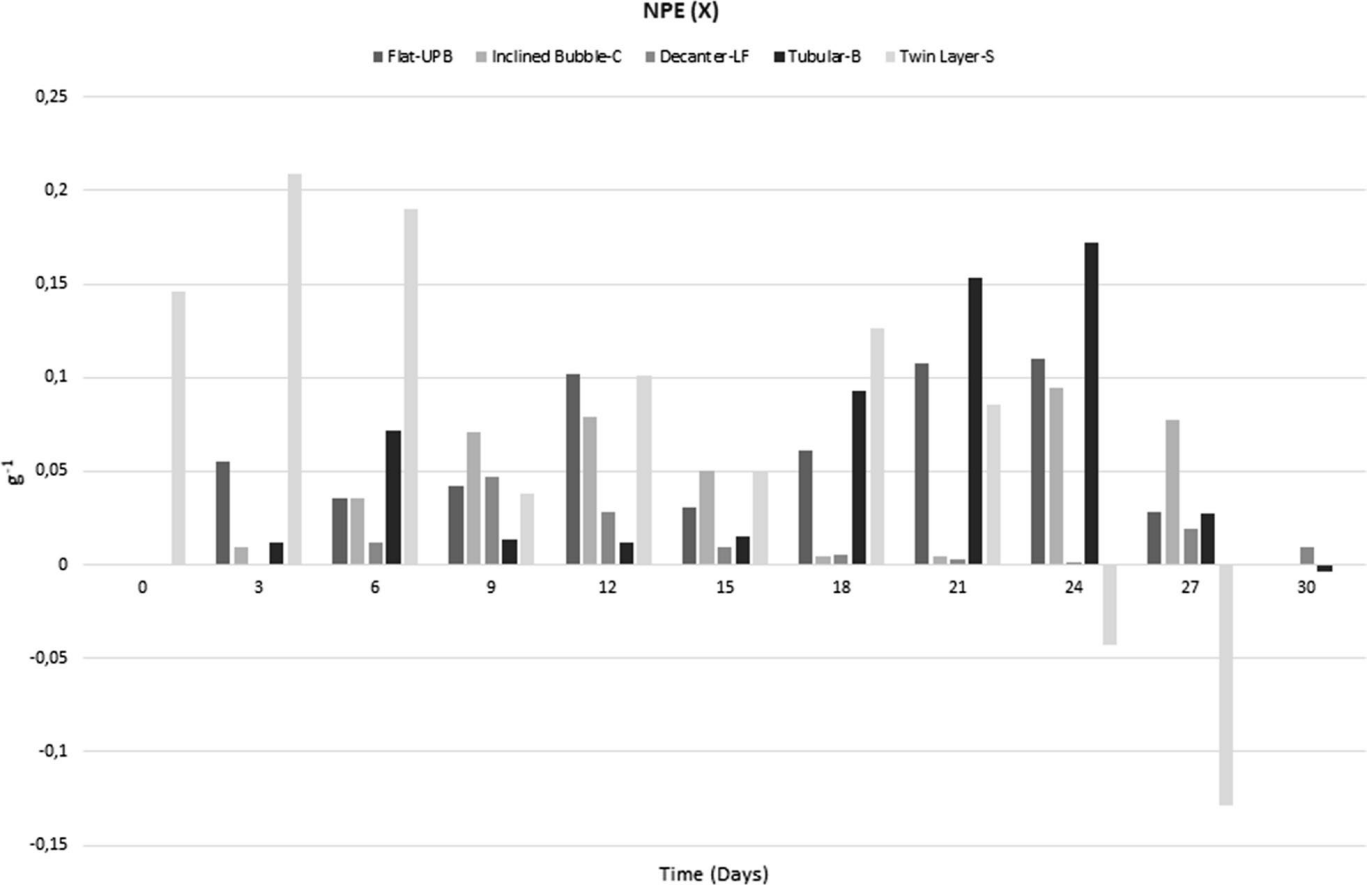
Normalized photosynthesis efficiency (NPE) in different photobioreactors during 30th days

When the normalized photosynthetic efficiency was calculated, the Decanter-LF showed the lowest level of NPE(*x*), that never exceeded 0.096 g^−1^. The Flat-UPB and the Inclined Bubble-C showed a similar maximum level of NPE (0.109 g^−1^ and 0.094 g^−1^, respectively). In the Tubular-B, NPE was lower than 1 g^−1^ until day 15; then it increased, with a maximum value of 0.188 g^−1^(Fig. 6).

In contrast, the Twin Layer-S, showed higher values of NPE during the first nine days of the experiment, resulting in a maximum of 0.208 g^−1^ on day 6; then the value decreased to about 0.1 g^−1^ (Fig. 6).

## Discussion

For the experiment, a light intensity of 100 μmol photons m^−2^ s ^−1^ was chosen because *G. sulphuraria* generally grows at low light intensities in the natural environment (Pinto et al. 2007; Eren et al. 2018) and also showed promising results in both liquid and immobilized cultivation systems with respect to its physiology and in relation to applications in biotechnology (e.g. Sano et al. 2001; Oesterhelt et al. 2007; Carbone et al. 2020). For the latter, exposition at this light intensity leads to an increase of phycobiliprotein production: Carbone et al. (2020) e.g. showed, in an experiment with a Twin Layer-S using different light intensities that 100 μmol photons m^−2^ s^−1^ was the optimal light intensity for production of phycobiliproteins, also Hirooka and Miyagishima (2016) obtained good production of phycocyanin at this light intensity in a suspended cultivation system using hot spring water supplemented with NH_4_^+^ as culture medium.

By comparing growth, productivity and photosyntesis performances, the Decanter LF showed the lowest level of biomass growth and photosynthetic performance, despite it is the most common system used for *G. sulphuraria* growth (Iovinella et al. 2018; Carfagna et al. 2018) it. Indeed, it was placed on a plate shaker; the absence of bubbling didn’t allow a good mixing of the culture for gas exchange, although the Decanter ensures a good mixing of nutrients around each cell surface (Rodriguez-Maroto et al. 2005; Mata et al. 2010).

In literature, better performances are commonly reported for microalgae in the Inclined Bubble-C and the Flat-UPB than in the Tubular-B. For example, Olivieri et al. (2011) showed that the green alga *Stichococcus bacillaris* grows better in the Inclined Bubble-C than in the Tubular-B; and De Vree et al. (2015) reported that *Nannochloropsis *sp*.* achieved higher biomass concentrations and enhanced photosynthesis performance in a flat panel cultivation system very similar to the Flat-UPB compared to other cultivation systems including a Tubular-B. Also, a number of studies found very high biomass levels were obtained with different microalgal genera such as *Nannochloropsis*, *Chlorococcum*, *Scenedesmus* and *Arthrospira* in a Flat UPB (Zhang et al. 2002; Koller et al. 2018; Hu et al. 1998; De Vree et al. 2015; Safafar et al. 2016; Tredici and Zitelli 1997).

In our experiments, *G. sulphuraria* had better performances in the Tubular-B among the suspended cultivation systems tested; these differences are probably linked to the particular physiology of this microalga. Indeed, *G. sulphuraria* is an extremophile organism that can survive in the dark up to five months (Gross et al. 1998) achieving very high biomass densities under heterotrophic conditions (Gross and Schnarrenberger 1995; Graverholt and Eriksen 2007; Eriksen 2018; Sloth et al. 2006). Generally, heterotrophy is not typical for red algae, and presumably, this is a strategy of *G.sulphuraria* to survive in extreme environments (Gross et al. 1998; Gaignard et al. 2019).

Therefore, the high illumination area of the Flat-UPB and high radial macroscopic circulation of the Inclined Bubble-C represent a drawback for an organism that lives in a cryptoendolithic condition, under which light is scarce or absent for days (Thangaraj et al. 2011; Gross et al. 1998; Janssen et al. 2003).

The Tubular-B has a low radial macroscopic circulation that causes a shadow effect, due to external microalgal biomass that capture most of the incident light, thus creating a low-light environment for inner cells of the suspension (González-Camejo et al. 2019; Hu et al. 1998; Kiperstok et al. 2017; Zuccaro et al. 2020; Carbone et al. 2019). In this way a condition similar to the endolithic state is generated.

Whereas the Inclined Bubble- C displayed lower growth and photosynthetic performance than the Tubular-B, the Flat-UPB had similar growth performance but lower photosynthetic activity than the Tubular-B.

The Tubular-B and the Flat-UPB had high chlorophyll contents, and as reported in the literature, this is directly connected to the photochemical performance of PSII, and, as a consequence, of photosynthetic activity and indirectly also to growth performance (Schreiber et al. 1998; Zuccaro et al. 2019).

However, algae grown in the Flat-UPB revealed higher percentage levels of carotenoids compared to those grown in the Tubular-B, indicating a stressful condition of the alga. Indeed, carotenoids perform an essential photoprotective role by quenching the triplet state chlorophyll molecules, scavenging toxic oxygen species formed during light stress, dissipating harmful excess excitation energy under light stress (Pisal and Lele 2005; Galasso et al. 2017; Takaichi 2011; González-Fernández et al. 2012; Sosa-Hernández et al. 2019; Sun et al. 2016).

Moreover, despite the good growth performance of the Flat UPB, the productivity is lower than that in the Tubular-B. Indeed, normalized photosynthesis performance is lower in the Flat-UPB.

Although the Tubular-B seems to be the best of the different suspended cultivation systems tested, the results obtained in this system are not comparable with the Twin Layer-S, in which *G. sulphuraria* exhibited best growth, photosynthetic performance and productivity. This result is not surprising: in natural environments, these microalgae generally live attached to substrates like soil or rocks and the Twin Layer-S partly reproduces conditions similar to the natural habitat of this species (Li et al. 2017; Melkonian and Podola 2010; Moreno Osorio 2018).

Moreover, in the Twin Layer-S the lower cell layers of the biofilm are permanently shaded by the upper cell layers due to immobilization of the cells, thus minimizing photoinhbition (Gross et al. 1998; Schultze et al. 2015; Piltz and Melkonian 2018; Langenbach and Melkonian 2019; Kim et al. 2019). In consequence, *G. sulphuraria* achieves high growth and photosynthetic performance also at light intensities that inhibit growth and photosynthetic performance in suspended cultures, such as 200 μmol photons m^−2^ s^−1^ (Carbone et al. 2020).

Eventually, Twin Layer-S offers also some economics advantages for mass cultures of *G. sulphuraria* (Carbone et al. 2017a; Podola et al. 2017; Pierobon et al. 2018; Zhuang et al. 2018). Many high costs linked to suspended cultivation systems are eliminated: for example, the biomass is harvested directly by scraping, without a preconcentration step; there are lower water consumption and space utilization. Furthermore, the system is modular, thus easily scalable. However, in comparison with submerged photobioreactor, which have been sufficiently tested and analysed also at pilot and industrial scale, the Twin-layer-S has still to be completely validated at a relevant and demonstrative scale. Thus, while techno-economic analysis of closed photobioreactor are already available in literature, an representative and meaningfull economic analysis of the Twin-layer-S has still to be performed.

## Data Availability

All data and materials are available.
